# The immune status, oxidative and epigenetic changes in tissues of turkeys fed diets with different ratios of arginine and lysine

**DOI:** 10.1038/s41598-021-95529-y

**Published:** 2021-08-05

**Authors:** Katarzyna Ognik, Dariusz Mikulski, Paweł Konieczka, Bartłomiej Tykałowski, Magdalena Krauze, Anna Stępniowska, Anna Nynca, Jan Jankowski

**Affiliations:** 1 Department of Biochemistry and Toxicology, Faculty of Animal Sciences and Bioeconomy, University of Life Sciences in Lublin, Akademicka 13, 20-950 Lublin, Poland; 2 Department of Poultry Science and Apiculture, Faculty of Animal Bioengineering, University of Warmia and Mazury in Olsztyn, Oczapowskiego 5, 10-719 Olsztyn, Poland; 3 Department of Poultry Diseases, Faculty of Veterinary Medicine, University of Warmia and Mazury in Olsztyn, Oczapowskiego 13/14, 10-719 Olsztyn, Poland; 4 Laboratory of Molecular Diagnostics, Faculty of Biology and Biotechnology, University of Warmia and Mazury in Olsztyn, Prawocheńskiego 5, 10-720 Olsztyn, Poland

**Keywords:** Animal physiology, Biochemistry, Immunology

## Abstract

In the present experiment, it was assumed that the appropriate dietary ratio of arginine (Arg) to lysine (Lys) can improve the immune status and growth performance of turkeys. The aim of this study was to evaluate the effects of two inclusion rates of Arg relative to Lys in turkey diets with Lys content consistent with National Research Council (NRC) recommendations or 10% higher on the immune status of birds and indicators of protein and DNA damage due to oxidation, nitration or epigenetic changes. Another goal was to determine which dietary Arg:Lys ratio stimulates the immune response of turkeys vaccinated against *Ornithobacterium rhinotracheale*. The experiment was performed on 576 turkeys randomly assigned to four groups with two levels of Lys (low = NRC recommendation or high = NRC + 10%) and two levels of Arg (95% or 105% Arg relative to the content of dietary Lys). It was found that the Lys content of turkey diets should be 10% higher than that recommended by the NRC and combined with the higher Arg level (105% of Lys). Although the above Arg:Lys ratio did not improve the growth performance of birds, it stimulated their immune system and reduced protein nitration as well as protein and DNA oxidation.

## Introduction

One of the challenges facing the modern poultry industry is increased risk of metabolic disorders caused by intensive farming as well as decreased resistance to infections and adverse environmental factors. Turkey diets with a balanced amino acid profile play a key role in maintaining good health and optimizing the growth potential of birds. Lysine (Lys) and arginine (Arg) are amino acids limiting the biological value of dietary protein in turkey diets. The optimal inclusion levels and quantitative ratios of Lys to Arg in turkey diets need to be established due to considerable differences between the recommendations of the NRC^1^ and breeding companies^2^, which stir much controversy. According to NRC guidelines^1^, diets fed to growing turkeys in the first 4 weeks of rearing should contain 1.60% Lys and 1.60% Arg, compared with 1.76% and 1.80%, respectively, recommended by breeding companies^2^. Similar differences in the amino acid requirements of turkeys can also be observed in later growth stages. The same applies to the optimal Arg:Lys ratio, since Arg should account for 90–100% of Lys content or 102–105% of Lys content according to the NRC^1^ and BUT^2^, respectively. In the concept of the "ideal protein" for turkeys in the first 4 weeks of life, Boling and Firman^3^ recommend 105% Arg over Lys, but they do not report Met content but only the sum of sulfur amino acids. Previous research shows that the dietary Arg:Lys ratio can significantly affect the immune and antioxidant status of birds^4–6^. Arginine is a substrate for the synthesis of intracellular nitric oxide (NO), and NO levels in the body are determined by the bioavailability of Arg. Nitric oxide plays an important role in the immune response because it participates in pathogen killing and initiates the signaling pathways that lead to the full expression of the immune response. Lysine and Arg share the same cellular transport systems, therefore the availability of dietary or extracellular Lys can modulate the entry of Arg into leukocytes and, consequently, NO synthesis by arginase and iNOS^7^. Closs et al.^8^ demonstrated that increasing extracellular Lys concentrations reduced intracellular Arg concentrations and NO synthesis in activated macrophages in a dose-dependent manner. According to many authors, NO synthesis can be limited by high intracellular Lys concentrations because Lys is capable of inhibiting arginase activity^9–11^. It should be stressed, however, that enhanced NO synthesis in cells can induce the oxidation of biologically important molecules and compromise immunity in birds. Grisham et al.^12^ and Hallemeesch et al.^13^ found that NO exerted immunostimulatory and antioxidant effects at low concentrations and during short-term exposure.

Our previous studies revealed that in turkey diets with Lys content consistent with NRC recommendations^1^, the optimal inclusion rate of Arg is 100% Lys, and methionine (Met) level should be higher than that recommended by the NRC^1^ to improve the growth performance and the immune and antioxidant status of birds. It was found that in diets with Lys content consistent with NRC recommendations^1^, the inclusion rate of Arg set at 90% Lys compromised the growth performance and the immune and antioxidant status of young turkeys during the entire rearing period. Diets with high Arg content (110% Lys) contributed to an increase in the proportion of breast muscles in the final BW of turkeys and did not induce the oxidation of lipids, proteins or DNA, but promoted undesirable protein nitration and an increase in thyroxine levels^14–16^. In another experiment conducted by our research team, where turkeys were fed diets with high Lys content (close to the recommendations of breeding companies^2^), an increase in the inclusion rate of Arg to 110% Lys increased the protein content of breast muscles, but did not improve the growth performance or the immune status of birds. A decrease in dietary Arg content to 90% Lys did not compromise performance only when the inclusion level of Met was increased to 45% Lys^17^. Since our studies of turkeys fed high-Lys diets revealed that low Arg concentration (90% Lys) stimulated the immune system, the question arose as to whether such an Arg:Lys ratio would be sufficient to effectively mobilize the immune system of birds in response to infection. According to some authors, a higher dietary Arg:Lys ratio than that recommended by the NRC^1^ supports the immune system of vaccinated or infected birds^18–23^. Our earlier experiment demonstrated that in turkeys fed high-Lys diets and infected with *C. perfringens*, Arg content can be decreased to 90% Lys provided that Met content is increased to 45% Lys. The above dietary amino acid ratios minimized oxidative processes and epigenetic alterations in important molecules in the intestinal wall and blood of turkeys, helped maintain intestinal barrier integrity, and exerted a beneficial influence on the metabolism and growth performance of birds^24^. The results of our previous studies also indicate that an increase in the dietary content of Met in excess of the levels recommended by NRC^1^ can boost immunity and improve the antioxidant status and growth performance of turkeys, including at the time of vaccination or infection^25–28^.

In view of these findings, which clearly show that the inclusion levels of Met in turkey diets should be higher than those recommended by the NRC^1^, it was assumed that the appropriate dietary ratio of Arg to Lys can improve the immune status and growth performance of turkeys. The aim of this study was to evaluate the effects of two inclusion rates of Arg relative to Lys (95% and 105%) in turkey diets with Lys content consistent with NRC recommendations^1^ or 10% higher on the immune status of birds and indicators of protein and DNA damage due to oxidation, nitration or epigenetic changes. Another goal was to determine which dietary Arg:Lys ratio stimulates the immune response of turkeys.

## Results

### Effect of dietary Arg:Lys ratios

The applied dietary treatments had no effect on the body weight (BW) of female turkeys or feed conversion ratio (FCR) throughout the experiment. Only in week 8, birds fed diets with the lower Arg level (95% Lys) had lower BW (*P* = 0.021) than those receiving diets with the higher Arg content (105% Lys) (Table 1).

**Table 1:** Body weights (BW) and the feed conversion ratio (FCR) in turkeys fed diets with different Arg:Lys ratios.

Item	BW (kg)				FCR (g/g)	Mortality (%)
	4 w	8 w	12 w	16 w	1–16 w	1–16 w
**Treatment**^**a**^						
L_L_A_L_	1.16	4.14	8.28	11.18	2.371	6.25
L_L_A_H_	1.18	4.21	8.39	11.35	2.376	6.25
L_H_A_L_	1.15	4.06	8.31	11.35	2.360	4.86
L_H_A_H_	1.17	4.25	8.43	11.40	2.381	6.25
SEM	0.006	0.029	0.036	0.049	0.008	ND
**Lysine (Lys)**						
Low (_L_)	1.17	4.18	8.33	11.27	2.373	6.25
High (_H_)	1.16	4.16	8.37	11.37	2.370	5.56
**Arginine (Arg)**						
Low (_L_)	1.16	4.10^b^	8.29	11.26	2.365	5.56
High (_H_)	1.18	4.23^a^	8.41	11.37	2.378	6.25
***P* value**						
Lys	0.286	0.686	0.612	0.302	0.854	ND
Arg	0.086	0.021	0.124	0.270	0.460	ND
Lys × Arg	0.639	0.235	0.979	0.552	0.666	ND

^a^L_L_A_L_ diet with low Lys and low Arg levels; L_L_A_H_ diet with low Lys and high Arg levels; L_H_A_L_ diet with high Lys and low Arg levels; L_H_A_H_ diet with high Lys and high Arg levels.

Diets with the higher Lys level (NRC + 10%) had no effect on the percentages of CD4+ and CD8 + cells, but contributed to an increase in the percentage of IgM+  B cells (*P* = 0.008) in the spleen (Table 2), and to a decrease in the levels of CRP (*P* = 0.034), PC (*P* < 0.001), 8-OHdG (*P* = 0.002) and Casp 3 (*P* = 0.008) in the small intestinal wall (Table 3), and a decrease in the plasma levels of 3-NT (*P* < 0.001) in 16-week-old birds (Table 4).

**Table 2:** Percentages of peripheral blood T cell and B cell subpopulations in turkeys at 9 weeks of age.

	Spleen				Blood			
	CD4+	CD8+	CD4+CD8+	IgM+	CD4+	CD8+	CD4+CD8+	IgM+
**Treatment**^**1**^								
L_L_A_L_	35.21	22.33^ab^	1.085	13.66	13.39^b^	1.624	0.171	5.923
L_L_A_H_	34.10	25.98^ab^	1.258	15.03	18.60^a^	1.856	0.185	6.058
L_H_A_L_	33.70	27.99^a^	1.275	15.38	15.86^ab^	1.841	0.218	6.005
L_H_A_H_	35.69	20.02^b^	1.270	17.94	14.26^b^	1.580	0.150	5.605
SEM	0.938	1.067	0.057	0.477	0.625	0.086	0.012	0.109
**Lysine (Lys)**								
Low (_L_)	34.66	24.15	1.171	14.34^b^	15.99	1.740	0.178	5.990
High (_H_)	34.69	24.00	1.273	16.66^a^	15.06	1.711	0.184	5.805
**Arginine (Arg)**								
Low (_L_)	34.46	25.16	1.180	14.52^b^	14.62	1.733	0.194	5.964
High (_H_)	34.89	23.00	1.264	16.48^a^	16.43	1.718	0.167	5.831
***P* value**								
Lys	0.985	0.939	0.395	0.008	0.396	0.868	0.820	0.406
Arg	0.824	0.269	0.480	0.023	0.105	0.935	0.270	0.551
Lys × Arg	0.434	0.005	0.455	0.469	0.004	0.170	0.101	0.233

^1^L_L_A_L_ diet with low Lys and low Arg levels; L_L_A_H_ diet with low Lys and high Arg levels; L_H_A_L_ diet with high Lys and low Arg levels; L_H_A_H_ diet with high Lys and high Arg levels.

^a,b^Values in same column with no common superscripts denote a significant difference (P ≤ 0.05).

**Table 3:** Blood immunological and redox parameters of turkeys at 9 weeks of age.

	IgY µg/mL	IgA µg/mL	Casp 8 ng/mL	Casp 3 ng/mL	IL-6 pg/mL	TNF pg/mL	Cp U/L	CRP mg/L	OGG1 ng/mL	APEX 1 ng/L	3-NT ng/mL	8-OHdG ng/mL	PC nmol/mg protein
**Treatment**^**1**^													
L_L_A_L_	349	51.86	4.068	1.494	12.33	13.29	1.206	1.274	13.13	637.1	43.52	0.228	1.483
L_L_A_H_	328	55.31	4.324	1.555	15.08	10.33	1.289	1.380	13.33	670.0	34.77	0.117	1.480
L_H_A_L_	345	58.25	3.901	1.585	15.23	11.58	1.265	1.610	13.61	641.9	39.44	0.221	1.427
L_H_A_H_	365	53.00	4.159	1.737	13.07	12.02	1.234	1.735	12.99	623.0	31.89	0.203	1.496
SEM	8.545	1.368	0.127	0.083	1.176	0.750	0.033	0.094	0.402	13.85	1.595	0.017	0.092
**Lysine (Lys)**													
Low (_L_)	338	53.59	4.196	1.525	13.71	11.81	1.248	1.327	13.23	653.5	39.15	0.176	1.481
High (_H_)	355	55.62	4.030	1.661	14.15	11.80	1.249	1.673	13.30	632.4	35.67	0.212	1.461
**Arginine (Arg)**													
Low (_L_)	347	55.05	3.985	1.540	13.78	12.43	1.235	1.442	13.37	639.5	41.48^a^	0.224	1.455
High (_H_)	346	54.16	4.242	1.646	14.08	11.18	1.262	1.557	13.16	646.5	33.33^b^	0.163	1.488
***P* value**													
Lys	0.335	0.461	0.529	0.432	0.857	0.996	0.984	0.071	0.936	0.463	0.241	0.230	0.918
Arg	0.967	0.745	0.332	0.540	0.904	0.417	0.701	0.536	0.805	0.806	0.009	0.057	0.865
Lys × Arg	0.249	0.122	0.998	0.793	0.321	0.275	0.409	0.959	0.628	0.369	0.838	0.160	0.855

IgA: immunoglobulin A; IgY: immunoglobulin Y; Casp 3: caspase 3; Casp 8: caspase 8; IL-6: interleukin 6; TNF-α: tumor necrosis factor alpha; Cp: ceruloplasmin; CRP: C-reactive protein; OGG1: oxoguanine glycosylase; APEX-1: endonuclease 1; 3-NT: 3- nitrotyrosine; 8-OHdG: 8*-*hydroxydeoxyguanosine; PC: protein carbonyl.

^1^L_L_A_L_ diet with low Lys and low Arg levels; L_L_A_H_ diet with low Lys and high Arg levels; L_H_A_L_ diet with high Lys and low Arg levels; L_H_A_H_ diet with high Lys and high Arg levels.

^a,b^Values in same column with no common superscripts denote a significant difference (P ≤ 0.05).

**Table 4:** Blood immunological and redox parameters of turkeys at 16 weeks of age.

	IgY µg/mL	IgA µg/mL	Casp 8 ng/mL	Casp 3 ng/mL	IL-6 pg/mL	TNF pg/mL	Cp U/L	CRP mg/L	OGG1 ng/mL	APEX 1 ng/L	3-NT ng/mL	8-OHdG ng/mL	PC nmol/mg protein
**Treatment**^**1**^													
L_L_A_L_	382	71.95	4.938	2.238	10.53	8.486	1.367	2.659	9.359^b^	633	51.19	0.473	0.847^ab^
L_L_A_H_	386	62.25	4.534	1.929	10.14	8.476	1.191	2.043	13.28^a^	692	35.44	0.360	0.748^b^
L_H_A_L_	349	61.04	4.265	2.044	9.091	8.227	1.247	2.003	10.50^ab^	642	31.28	0.320	0.758^b^
L_H_A_H_	362	62.10	4.404	1.741	10.13	8.948	1.248	1.988	11.17^ab^	682	24.82	0.301	0.986^a^
SEM	7.65	1.515	0.121	0.096	0.648	0.296	0.044	0.123	0.438	12.92	2.183	0.032	0.039
**Lysine (Lys)**													
Low (_L_)	384	67.10	4.736	2.083	10.33	8.481	1.279	2.351	11.32	662.3	43.32^a^	0.416	0.797
High (_H_)	356	61.57	4.334	1.893	9.610	8.588	1.247	1.995	10.83	662.1	28.05^b^	0.310	0.872
**Arginine (Arg)**													
Low (_L_)	365	66.50	4.602	2.141	9.808	8.357	1.307	2.331	9.928	637.4	41.23^a^	0.396	0.802
High (_H_)	374	62.18	4.469	1.835	10.13	8.712	1.219	2.016	12.22	687.0	30.13^b^	0.330	0.867
***P* value**													
Lys	0.070	0.051	0.101	0.325	0.597	0.864	0.727	0.142	0.521	0.993	< 0.001	0.098	0.319
Arg	0.564	0.123	0.580	0.119	0.810	0.568	0.339	0.192	0.005	0.061	< 0.001	0.296	0.390
Lys × Arg	0.791	0.058	0.262	0.987	0.601	0.557	0.332	0.212	0.039	0.711	0.103	0.449	0.035

^1^L_L_A_L_ diet with low Lys and low Arg levels; L_L_A_H_ diet with low Lys and high Arg levels; L_H_A_L_ diet with high Lys and low Arg levels; L_H_A_H_ diet with high Lys and high Arg levels.

^a,b^Values in same column with no common superscripts denote a significant difference (P ≤ 0.05).

In comparison with the lower Arg concentration (95% Lys), the higher inclusion rate of Arg (105% Lys) led to an increase in the percentage of IgM+ B cells (*P* = 0.023) in the spleen (Table 2), and a decrease in the levels of 3-NT in the small intestinal wall (*P* = 0.030) (Table 5) and blood plasma of turkeys (*P* = 0.009 and *P* < 0.001, respectively) (Tables 3 and 4). The levels of 8-OHdG decreased in the blood plasma and small intestinal wall (*P* = 0.014 and *P* = 0.015, respectively) of 9-week-old turkeys fed diets with the higher Arg content (105% Lys) (Tables 3 and 5). The higher inclusion rate of Arg (105% Lys) increased DNA methylation (*P* = 0.045) in the small intestinal wall (Table 5) but not in the blood plasma of turkeys at 9 and 16 weeks of age.

**Table 5:** Immunological, redox parameters and global methylation DNA in the intestinal wall of turkeys at 9 weeks of age.

	IgY µg/g	IgA µg/g	Casp 8 ng/g	Casp 3 ng/g	IL-6 pg/g	TNF pg/g	Cp U/g	CRP mg/g	OGG1 ng/g	APEX 1 ng/g	3-NT ng/g	8-OHdG ng/g	PC nmol/mg protein	% DNA methylation
**Treatment**^**1**^														
L_L_A_L_	3250	590.3	44.40	24.26	1469	620.1	0.014	0.029	91.14	5.111	125.57	88.82	0.877	43.95
L_L_A_H_	3113	551.1	40.80	22.26	1382	648.7	0.012	0.025	91.63	4.786	112.88	86.03	0.785	47.22
L_H_A_L_	3096	552.9	39.70	19.76	1371	667.0	0.012	0.024	84.17	4.851	117.34	83.75	0.356	41.42
L_H_A_H_	3322	557.4	39.76	18.27	1379	666.1	0.011	0.020	90.45	4.976	79.89	73.14	0.364	49.26
SEM	71.86	10.77	1.317	0.814	32.86	15.13	0.0004	0.001	1.606	0.093	6.086	1.614	0.053	1.372
**Lysine (Lys)**														
Low (_L_)	3181	570.7	42.60	23.26^a^	1426	634.4	0.013	0.027^a^	91.39	4.948	119.22	87.42^a^	0.831	45.59
High (_H_)	3209	555.1	39.73	19.02^b^	1375	666.5	0.011	0.022^b^	87.31	4.913	98.61	78.45^b^	0.360	45.34
**Arginine (Arg)**														
Low (_L_)	3173	571.6	42.05	22.01	1420	643.5	0.013	0.026	87.66	4.981	121.45^a^	86.29^a^	0.617	42.68^b^
High (_H_)	3217	554.2	40.28	20.27	1381	657.4	0.012	0.023	91.04	4.881	96.38^b^	79.58^b^	0.575	48.24^a^
***P* value**														
Lys	0.854	0.482	0.293	0.008	0.464	0.310	0.122	0.034	0.211	0.856	0.071	0.002	< 0.001	0.925
Arg	0.766	0.434	0.513	0.247	0.559	0.658	0.231	0.143	0.296	0.606	0.030	0.014	0.535	0.045
Lys × Arg	0.227	0.326	0.499	0.864	0.483	0.638	0.727	0.973	0.370	0.247	0.270	0.136	0.463	0.397

^1^L_L_A_L_ diet with low Lys and low Arg levels; L_L_A_H_ diet with low Lys and high Arg levels; L_H_A_L_ diet with high Lys and low Arg levels; L_H_A_H_ diet with high Lys and high Arg levels.

IgA: immunoglobulin A; IgY: immunoglobulin Y; Casp 3: caspase 3; Casp 8: caspase 8; IL-6: interleukin 6; TNF-α: tumor necrosis factor alpha; Cp – ceruloplasmin; CRP: C-reactive protein; OGG1: oxoguanine glycosylase; APEX-1: endonuclease 1; 3-NT: 3- nitrotyrosine; 8-OHdG: 8*-*hydroxydeoxyguanosine; PC: protein carbonyl.

^a,b^Values in same column with no common superscripts denote a significant difference (P ≤ 0.05).

### Effect of dietary Arg:Lys ratios in vaccinated and non-vaccinated turkeys

Diets with the higher Lys level (NRC + 10%) had no effect on IgA gene expression in vaccinated turkeys, but increased IgA gene expression (*P* = 0.043) in the liver of non-vaccinated 6-week-old turkeys (Table 6). Vaccine-induced antibody titers against ORT in the blood serum of vaccinated 16-week-old turkeys were not affected by the higher Lys level (NRC + 10%) (Table 7). Neither low (95% Lys) nor high (105% Lys) Arg levels affected IgA gene expression (Table 6) or vaccine-induced antibody titers against ORT in the blood serum of vaccinated turkeys (Table 7).

**Table 6:** The mRNA expression of selected genes in the liver of vaccinated and non-vaccinated turkeys at 6 weeks of age.

	IgA		IL-6	
	Non-vaccinated	Vaccinated	Non-vaccinated	Vaccinated
**Treatment**^**1**^				
L_L_A_L_	0.013	0.006^ab^	< LOQ	< LOQ
L_L_A_H_	0.012	0.005^ab^	< LOQ	< LOQ
L_H_A_L_	0.010	0.004^b^	< LOQ	< LOQ
L_H_A_H_	0.009	0.007^a^	< LOQ	< LOQ
SEM	0.0008	0.0005		
**Lysine (Lys)**				
Low (_L_)	0.012^a^	0.005	–	–
High (_H_)	0.009^b^	0.006	–	–
**Arginine (Arg)**				
Low (_L_)	0.012	0.005	–	–
High (_H_)	0.010	0.006	–	–
***P* value**				
Lys	0.043	0.851	–	–
Arg	0.271	0.213	–	–
Lys × Arg	0.951	0.040	–	–

^1^L_L_A_L_ diet with low Lys and low Arg levels; L_L_A_H_ diet with low Lys and high Arg levels; L_H_A_L_ diet with high Lys and low Arg levels; L_H_A_H_ diet with high Lys and high Arg levels.

IgA: immunoglobulin A. IL-6: interleukin; < LOQ- below the limit of quantification.

^a,b^Values in same column with no common superscripts denote a significant difference (P ≤ 0.05).

**Table 7:** Vaccine-induced antibody titers against ORT in the blood serum of turkeys at 16 weeks of age.

	Non-vaccinated	Vaccinated
**Treatment**^**1**^		
L_L_A_L_	358.1	8632
L_L_A_H_	411.5	8699
L_H_A_L_	295.7	7411
L_H_A_H_	303.7	8689
SEM	21.22	466.5
**Lysine (Lys)**		
Low (_L_)	384.8^a^	8666
High (_H_)	299.7^b^	8050
**Arginine (Arg)**		
Low (_L_)	326.9	8021
High (_H_)	357.6	8694
***P* value**		
Lys	0.046	0.515
Arg	0.467	0.477
Lys × Arg	0.590	0.522

^1^L_L_A_L_ diet with low Lys and low Arg levels; L_L_A_H_ diet with low Lys and high Arg levels; L_H_A_L_ diet with high Lys and low Arg levels; L_H_A_H_ diet with high Lys and high Arg levels.

^a,b^Values in same column with no common superscripts denote a significant difference (P ≤ 0.05).

## Discussion

A few experiments have shown that the growth performance of turkeys is affected by the dietary inclusion levels and ratios of Arg and Lys^29,30^, and that an Arg:Lys ratio higher than 1:1^1^ improved productivity^30^. Our previous findings indicate that different dietary proportions of Arg relative to Lys (90%, 100% and 110% Lys) in diets with high Lys content, close to the recommendations of breeding companies^2^, have no influence on the growth rate of turkeys^14^. In another experiment, different concentrations of Arg (90%, 100% and 110% Lys) in low-Lys diets^1^ had no effect on the final BW of turkeys, except in the youngest birds (aged 1–8 weeks) where the lowest Arg level (90% Lys) decreased BW^17^. In the present study, where turkeys received diets with Lys content consistent with NRC recommendations^1^ or 10% higher, the dietary proportions of Arg relative to Lys (95% and 105% Lys) had no influence on the FCR or final BW. However, a decrease in BW was noted in young turkeys fed diets with the lower Arg level (95% Lys). Lower BWG in growing turkeys administered diets with low Arg content (90–95% Lys) and Lys content consistent with NRC recommendations^1^ or 10% higher can be attributed to the fact that young birds are characterized by a faster growth rate and higher Arg requirements than older birds^31^. Rapid growth in the first weeks of life is accompanied by a high metabolic rate, and Arg produces creatine which enhances the release of insulin-like growth factor 1 (IGF-1) in the muscles of birds^32,33^.

According to published research, Lys-deficient diets suppress the synthesis of proteins (including cytokines) and lymphocyte proliferation, decrease cell-mediated immunity and impair the antibody response, thus increasing disease incidence and mortality^34–37^. In the current study, the higher dietary inclusion rate of Lys (NRC + 10%) had no effect on the percentages of CD4^+^ and CD8^+^ cells, but induced an increase in the percentage of IgM^+^ B cells in the spleen of young turkeys. According to the literature, diets with increased concentrations of Met or Arg can be a major contributor to the synthesis of immune system proteins, including IgA and IgY^21,38^. The liver plays a unique role in much immunity and in the physiology of IgA in normal and disease states**.** In the present experiment, IgA gene expression in the liver was higher in young turkeys fed diets with the higher Lys level (NRC + 10%) than in those receiving diets with Lys content recommended by the NRC^1^, but it had no effect on total IgA and IgY levels in the blood plasma and intestinal wall. C-reactive protein plays an important role in recognizing self and foreign molecules, and leads to the activation of the adaptive immune system in the early stage of inflammation or infection by interacting with the complement and Fc receptors on phagocytes^8^. The decrease in CRP levels in the small intestinal wall of turkeys fed diets with the higher Lys content (NRC + 10%), noted in the present study, suggests that the immune system of turkeys effectively protected them against pathogens and infection. According to Calder and Yaqoob^39^, Arg is necessary for efficient proliferation of T and B cells. De Jonge et al.^40^ reported that Arg deficiency resulting from arginase overexpression in the small intestine impaired B cell proliferation in the lymphoid organs of transgenic mice. Therefore, a reduced number of B cells leaves the bone marrow and enters the lymphoid organs, thus considerably reducing the number of B cells in the spleen and lymph nodes and as well as serum IgM levels. In the current experiment, the higher dietary inclusion rate of Arg (105% Lys) induced an increase in the percentage of IgM^+^ B cells in the spleen of young turkeys, but it had no influence on IgY and IgA levels or the percentage of T cells in blood.

The appropriate Arg:Lys ratio in turkey diets affects not only the immune system but also redox reactions. High dietary Arg levels can enhance NO synthesis. Endogenous NO has immunostimulatory properties, but its excess concentrations in cells can stimulate the oxidation and nitration of biologically important molecules, and DNA oxidation^41^. Excess synthesis of NO may be suppressed by high concentrations of Lys in cells because Lys can inhibit the activity of arginase responsible for NO synthesis from Arg^9–11^. In the present study, the higher dietary inclusion levels of Lys (NRC + 10%) and Arg (105% Lys) desirably suppressed protein and DNA oxidation, as manifested by reduced levels of PC and 8-OHdG in the small intestinal wall and blood plasma of turkeys. The applied dietary treatments had no effect on the levels of DNA repair enzymes, which confirms that increased inclusion rates of Lys and Arg did not induce cell oxidation. In our previous study, graded dietary Arg levels (90%, 100% and 110%) relative to Lys content consistent with NRC recommendations^1^ had no influence on the oxidation of lipids, protein or DNA, but the highest inclusion rate of Arg (110% Lys) induced protein nitration^16^. However, in the current experiment, the higher dietary inclusion levels of Arg (105% Lys) and Lys (NRC + 10%) did not promote protein nitration or even inhibited these undesirable reactions. According to Yin et al.^42^, dietary Lys deficiency impairs amino acid metabolism and leads to cell apoptosis, thus increasing Casp 3 levels. In one of our previous experiments, neither a decrease in Arg content from 100 to 90% Lys nor its increase to 110% Lys in high-Lys diets^2^ affected Casp 3 or Casp 8 levels in turkeys^17^. In another study of turkeys fed low-Lys diets^1^, an increase in Arg content to 110% Lys increased Casp 3 levels^14^. In the present experiment, the higher Lys concentration (NRC + 10%) decreased Casp 3 levels, which indicates that this amino acid had a positive effect on cell apoptosis and redox reactions. According to Ulrey et al.^43^, amino acid metabolism (in particular Met, Lys and Arg) plays an important role in DNA methylation. In the present study, enhanced DNA methylation in the small intestinal wall of turkeys fed diets with increased Arg content (105% Lys) was undesirable and could be linked to Arg methylation^44^. Moreover, it could be a consequence of the direct effect of NO-modified compounds (which affect epigenetic regulations) on DNA.

Kubińska et al.^26^ demonstrated that turkeys that had been re-vaccinated twice against ORT with an inactivated vaccine and received diets with increased Met content (relative to NRC recommendations^1^), had considerably higher serum levels of vaccine-induced antibody titres. These studies show that the immune system of vaccinated turkeys can be additionally stimulated by the increased level of the amino acid. Literature data show that apart from Met, also Arg^6,14,20^ and Lys^14,17^ can stimulate the immune system. The present results do not corroborate the findings of Kubińska et al.^26^, because the higher dietary inclusion levels of Lys and Arg, relative to NRC recommendations^1^, had no influence on vaccine-induced antibody titres in turkeys vaccinated against ORT. According to Tykałowski et al.^28^, and Guiro and Koncicki^45^, once-vaccinated turkeys usually do not develop high levels of serum antibodies, which may explain the present results. In commercial turkeys, veterinarians quite often use a single vaccination against ORT, and then the titre of vaccine antibodies is lower and lasts less than after re-vaccination. Due to the fact that slaughter turkeys live much shorter than breeding turkeys, usually such protection is sufficient for them with low pressure of the field ORT. Kubińska et al.^26^ observed elevated IgA levels in turkeys vaccinated against ORT and fed diets with increased Met content. In the current experiment, a few days after vaccination against ORT, young turkeys receiving a diet with the higher inclusion rates of Lys (NRC + 10%) and Arg (105% Lys), were characterized by higher IgA gene expression in the liver than those fed a diet with the higher Lys level and the lower Arg level (95% Lys). Thus, the expression of IgA was influenced by an increased level of Arg in the diet. l-arginine plays an important role in maintaining immune homeostasis^46,47^. In studies with chickens, it has been established that increasing Arg in the diet results in an increase in the secretion of mucosal IgA^48^. The influence of Arg on the immunity of turkeys is evidenced by the increased expression of IgA in the liver, but no increased level of this immunoglobulin in the blood was recorded. There was also no effect of the increased Arg level on the other tested parameters of the immune system of turkeys. In our previous study of turkeys administered diets with Lys content consistent with NRC guidelines^1^ or higher, as recommended by BUT^2^, high Arg concentration (110% Lys) did not increase plasma IgA levels^13,16^.

## Conclusions

The results of this study indicate that the Lys content of turkey diets should be 10% higher than that recommended by the NRC^1^. The increased Lys content should be combined with the higher Arg level (105% of Lys content). Although the above Arg:Lys ratio did not improve the growth performance of birds, it stimulated their immune system (in particular the immune response following vaccination) and reduced protein nitration as well as protein and DNA oxidation.

## Materials and methods

### Experimental design and diets

The protocol for the study was approved by the Local Ethics Committee for Animal Experiments in Olsztyn, Poland (decision No. 82/2017), and the animals were cared for under guidelines comparable to those laid down by EU Directive 2010/63/EU. The study was carried out in compliance with the ARRIVE guidelines. One-day-old female Hybrid Converter turkeys were purchased from the Grelavi commercial hatchery in Kętrzyn. The experiment was performed on 576 turkeys that were randomly assigned to 32 pens of 18 birds each. The experiment had a completely randomized design with four dietary treatment groups, eight replicate pens per group and 18 birds per pen. The replicate pens were uniformly distributed in the house. Pens with a floor area of 4 m^2^ (2.0 m × 2.0 m) were bedded with wood shavings. The stocking density in the initial stage of rearing was 4.50 birds/m^2^. Environmental conditions, consistent with Hybrid requirements, were identical for all turkeys in the housing facility. They were controlled automatically and adjusted to the birds’ age. Throughout the experiment, all birds had free access to feed and water. The feed and drink lines were adjusted to the growth stage of turkeys.

The experimental diets were produced in a local feed mill under the direct supervision of a representative of the Department of Poultry Science and Apiculture, University of Warmia and Mazury in Olsztyn. According to the experimental procedure, basal diets were prepared for each of the four feeding periods, and their amino acid content was determined analytically (Table 8). Then the diets were mixed with the appropriate amounts of Lys, Met and Arg (Table 8). The actual levels of supplementary Lys, Arg and Met in experimental diets were obtained by adding supplementary L-Lys HCl, L-Arg HCl and DL-Met on top to the basal feed.

**Table 8:** Ingredient composition and nutrient content of basal diets (g/100 g, as-fed basis) fed to turkeys.

Item	Feeding period, weeks			
	1–4	5–8	9–12	13–16
**Ingredients**				
Wheat	56.454	56.576	64.037	64.472
Maize	–	–	–	10.000
Soybean meal, 48% CP	25.380	27.722	21.121	9.324
Rapeseed meal	4.566	5.000	6.000	7.000
Potato protein	5.000	–	–	–
Soybean oil	0.892	3.088	4.725	4.371
Maize gluten meal	3.000	2.769	–	2.000
Sodium bicarbonate	0.200	0.20	0.100	0.200
Sodium chloride	0.158	0.161	0.217	0.095
Limestone	2.046	1.766	1.391	1.075
Monocalcium phosphate	1.707	1.390	1.409	0.558
l lysine HCl	–	0.317	0.347	0.300
dl methionine	0.195	0.231	0.163	0.072
l-threonine	0.052	0.131	0.139	0.182
Choline chloride	0.10	0.100	0.100	0.100
Vitamin-mineral premix^a^	0.25	0.250	0.250	0.250
Titanium oxide	–	0.300	–	–
**Calculated nutrient content**^**b**^				
Metabolizable energy, kcal/kg	2800	2900	3000	3150
Crude protein	26.35 (26.79)	24.0 (24.33)	20.3 (20.31)	17.0 (17.35)
Arginine	1.52 (1.50)	1.42 (1.38)	1.21 (1.25)	0.92 (0.89)
Lysine	1.30 (1.23)	1.35 (1.36)	1.20 (1.18)	0.90 (0.76)
Methionine	0.62 (0.61)	0.59 (0.56)	0.46 (0.45)	0.35 (0.38)
Methionine + cysteine	1.09 (1.06)	1.03 (0.97)	0.85 (0.83)	0.71 (0.72)
Threonine	1.05 (1.03)	0.97 (0.94)	0.84 (0.83)	0.76 (0.78)
Calcium	1.25	1.10	0.95	0.65
Available phosphorus	0.65	0.55	0.47	0.32
**Amino acids added to basal diets**				
L_L_A_L_^c^				
l-Lysine HCl^d^	0.47	0.18	0.15	0.30
l-Arginine HCl	0.02	0.05	–	0.06
dl-Methionine	–	0.03	0.06	–
L_L_A_H_				
l-Lysine HCl	0.47	0.18	0.15	0.30
l-Arginine HCl	0.18	0.20	0.12	0.16
dl-Methionine	–	0.03	0.06	–
L_H_A_L_				
l-Lysine HCl	0.67	0.37	0.32	0.43
l-Arginine HCl	0.17	0.19	0.11	0.16
dl-Methionine	–	0.03	0.06	–
L_H_A_H_				
l-Lysine HCl	0.67	0.37	0.32	0.43
l-Arginine HCl	0.35	0.35	0.25	0.27
dl-Methionine	–	0.03	0.06	–

^a^Provided per kg diet (feeding periods: weeks 0–4, 5–8, 9–12 and 13–16): mg: retinol 3.78, 3.38, 2.88, and 2.52; cholecalciferol 0.13, 0.12, 0.10, and 0.09; a-tocopheryl acetate 100, 90, 80, and 70; vit. K3 5.8, 5.6, 4.8, and 4.2; thiamine 5.4, 4.7, 4.0, and 3.5; riboflavin 8.4, 7.5, 6.4, and 5.6; pyridoxine 6.4, 5.6, 4.8, and 4.2; cobalamin 0.032, 0.028, 0.024, and 0.021; biotin 0.32, 0.28, 0.24, and 0.21; pantothenic acid 28, 24, 20, and 18; nicotinic acid 84, 75, 64, and 56; folic acid 3.2, 2.8, 2.4, and 2.1; Fe 64, 60, 56 and 48; Mn 120, 112, 96, and 84; Zn 110, 103, 88, and 77; Cu 23, 19, 16, and 14; I 3.2, 2.8, 2.4, and 2.1; Se 0.30, 0.28, 0.24, and 0.21, respectively.

^b^The value in parentheses was determined analytically.

^c^L_L_A_L_, diet with low Lys and low Arg levels; L_L_A_H_, diet with low Lys and high Arg levels; L_H_A_L_, diet with high Lys and low Arg levels; L_H_A_H_, diet with high Lys and high Arg levels.

^d^l-Lysine HCl contained 780 g lysine/kg (Ajinomoto Eurolysine S.A.S). l-Arginine HCl contained 990 g arginine/kg (Ajinomoto Eurolysine S.A.S). dl-Methionine contained 990 g methionine/kg (MetAMINO; Evonik Degussa Gmbh).

Two dietary inclusion levels of Lys were analyzed, low (L_L_ = NRC) and high (L_H_ = NRC + 10%). In diets with the low level of Lys, l-Lysine HCl was added to the basal diet to obtain 1.60, 1.50, 1.30 and 1.00 g of Lys per 100 g of feed in four successive feeding periods, according to NRC guidelines^1^. l-Arginine HCl was added to the basal diet to obtain 95% and 105% Arg relative to the content of dietary Lys (low and high, A_L_ and A_H_ respectively). The effects of four experimental diets, with two levels of Lys and two levels of Arg (L_L_A_L_, L_L_A_H_, L_H_A_L_, L_H_A_H_), were compared in the study. The inclusion rate of Met in experimental diets was identical, and dl-Methionine was added to obtain 0.62, 0.59, 0.51 and 0.39 g of Met per 100 g of feed in four successive feeding periods. As a result, the Met:Lys ratio was 0.39 in experimental diets with the low Lys level (L_L_) and 0.35 in experimental diets with the high Lys level (L_H_), regardless of the stage of rearing. Throughout the experiment, the birds had free access to feed and water. The diets were offered as crumbles (days 1–28) and pellets.

### Vaccine and vaccination

At 34 days of age, half of the birds in each replicate were vaccinated against *Ornithobacterium rhinotracheale* (ORT) with Ornitin (ABIC Biological Laboratories Ltd., Israel). The vaccine was administered subcutaneously following the manufacturer's instructions.

### Growth trial and sample collection

The body weights (BW) of birds were recorded and calculated on a pen basis. The feed conversion ratio (FCR; kg of feed/kg of body weight gain, BWG) for the experimental period was calculated on a pen basis from BWG and feed consumption. Mortality rates and causes were recorded daily, and the weights of dead birds were used to adjust the average FCR.

Blood samples were collected at 9 and 16 weeks of age from the wing vein. For all analysis besides serological analysis at 9 and 16 weeks blood was collected from 8 birds in each group (one bird per replicate) with BW similar to the treatment average. While for serological analysis blood samples were collected (into sterile tubes with clot activator) at 16 weeks from 23 birds per treatment that were not vaccinated and 23 birds per treatment that were vaccinated against ORT. Immediately after collection, blood samples were aliquoted into test tubes containing EDTA K (for flow cytometry analyses) or heparin (for other analyses) as an anticoagulant. Blood samples for cytometric analyses were directly sent for isolation of mononuclear cells. While the samples for other analyses were centrifuged for 15 min at 380*g* and 4 °C, and the resulting plasma was stored at − 20 °C until analysis.

At 9 weeks of age, 8 turkeys from each treatment were sacrificed by cervical dislocation, and the abdominal cavity was opened for the collection of ileal (middle-ileum) tissues and spleen samples (not vaccinated against ORT). Liver samples were collected from 8 birds per treatment (determined separately for non-vaccinated and vaccinated birds).

### Laboratory analyses

Peripheral blood mononuclear cells (PBMCs) were isolated according to a previously described procedure by Koncicki et al.^49^. The isolation of mononuclear cells from the spleen and the determination of the percentages of CD4+ and CD8+ T cell and IgM^+^ B cell subpopulations in PBMCs and spleen were carried out as described by Kubińska et al.^50^.

DNA was isolated from the intestinal wall using QIAGEN kits. Epigenetic changes in the blood and intestinal wall of turkeys were determined by analyzing global DNA methylation (methylome) with the use of Sigma Aldrich diagnostic kits. The levels of 8*-*hydroxydeoxyguanosine (8-OHdG), endonuclease 1 (APE-1) and oxoguanine glycosylase (OGG1) in the blood and intestinal wall of turkeys were determined using OxiSelect diagnostic kits (Cell Biolabs, Inc., San Diego, USA). OxiSelect diagnostic kits (Cell Biolabs, Inc., San Diego, USA) were also used to determine protein carbonyl (PC) and 3-nitrotyrosine (3-NT) derivatives as an indicator of the oxidation of amino acid residues. The levels of caspase 3 (Casp 3) and caspase 8 (Casp 8) were determined in the blood and intestinal wall of turkeys using an ELISA kit (Cell Biolabs, Inc. San Diego, USA). The plasma levels of C-reactive protein (CRP) were determined in an ELISA reader using assays from Elabscience Biotechnology Co., Ltd. (Houston, Texas, USA). The levels of ceruloplasmin (Cp) in the blood and jejunum of turkeys were determined using a Ceruloplasmin ELISA kit (Biomatik, Delaware, USA). The levels of total globulins and immunoglobulins IgA and IgY, tumor necrosis factor alpha (TNF-α), and interleukin 6 (IL-6) were determined in an ELISA reader using assays from Elabscience Biotechnology Co., Ltd. (Houston, Texas, USA).

Anti-ORT IgY titres were determined using a commercial immunoenzymatic ELISA kit (IDEXX Laboratories, USA) according to the manufacturer’s recommendations. The ELISA assay was performed using an epMotion 5075 LH automated pipetting system (Eppendorf), an Elx405 washer, and an Elx800 absorbance microplate reader (BioTek, USA).

### Analysis of mRNA transcript levels

The relative mRNA expression levels of genes encoding IgA and IL-6 were quantified in liver samples collected from turkeys 2 days after vaccination against ORT. The protocol of qRT-PCR analysis was described by Ognik et al.^24^. The liver samples were immediately frozen in liquid nitrogen. Subsequently, RNA was isolated using the GeneMATRIX Universal RNA Purification Kit (Eurx, Gdańsk, Poland), in accordance with the manufacturer’s instructions. The concentration of RNA was measured on a NanoDrop spectrophotometer (Nanodrop, NanoDrop Technologies, Wilmington, DE), and RNA integrity was verified on agarose gel under denaturing conditions. qPCR reactions were performed on a LightCycler 480 II apparatus (Roche Applied Science, CA, USA) using the SG qPCR Master Mix (Eurx, Gdańsk, Poland). Reaction conditions were as follows: (i) initial denaturation (one cycle at 95 °C for 10 min) and (ii) amplification (35 cycles of denaturation at 95 °C for 10 s, primer annealing at 58 °C for 10 s and DNA synthesis at 72 °C for 20 s). Endogenous control genes, β-Actin (ACTB) and Vimentins (VIM), were used to normalize gene expression data. The primers for the target and reference genes used in this study are presented in Table 9.

**Table 9:** Genes and primers used in the study.

Gene	Primer	Sequence (5ʹ–3ʹ)	Melting temperature (°C)	Product size (nt)
ACTB	Forward	TACCCCATTGAACACGGCAT	58	96
	Reverse	CTCCTCAGGGGCTACTCTCA		
VIM	Forward	GGAACAATGATGCCCTGC	58	145
	Reverse	GCAAAATTCTCCTCCATTTCAC		
IL-6	Forward	AAGGCGTGGATAGAGAAG	58	158
	Reverse	TGACAGATCGGTAACAGAG		
IgA	Forward	TCCGGGTTCACCTTCAGC	58	163
	Reverse	CTGTGCTCTGCCCGTTGT		

ACTB: B-actin; VIM: vimentin; IL-6: interleukin 6; IgA: immunoglobulin A.

### Statistical analysis

The results were analyzed statistically by two-way ANOVA using STATISTICA ver. 13 software (StatSoft Inc. 2013). The significance of differences between means was determined by Tukey’s test or the non-parametric Kruskal–Wallis test. The variability of data was expressed as standard deviation (σ) and the standard error of the mean (SEM). The results were regarded as statistically significant at *P* < 0.05.
